# Advocating for an open communication culture in perfusion and cardiothoracic community: a call to action

**DOI:** 10.1051/ject/2023041

**Published:** 2024-03-15

**Authors:** Salman Pervaiz Butt, Yasir Saleem, Bill Cook

**Affiliations:** 1 Perfusionist & ECMO Specialist, Cleveland Clinic Abu Dhabi PO BOX: 112412 United Arab Emirates; 2 Clinical Perfusionist, Department of CTVS, All India Institute of Medical Science-Rishikesh India; 3 Clinical Perfusionist, Perfusion Department, University Hospital of Leicester, Glenfield United Kingdom

## Abstract

This article advocates for an open communication culture in the perfusion and cardiothoracic community to enhance patient safety during surgery. All team members, including nurses, anesthesiologists, and perfusionists, should actively contribute their insights. Empowering perfusionists to voice concerns without fear of repercussions is crucial. Involvement in debriefs, root cause analyses and data management systems aids continuous improvement. A robust speak-up culture prevents unsafe practices and elevates perfusion care standards, leading to better patient outcomes.

## Discussion

Open communication is crucial in intraoperative cardiothoracic surgery to ensure patient safety and quality outcomes. Despite hierarchical structures, a well-functioning surgical team values input from all members, recognizing the valuable insights and observations they can provide. Nurses, anesthesiologists, perfusionists, and other staff members play essential roles in enhancing patient safety during surgery [1].

The WHO surgical safety checklist emphasizes communication and teamwork in the operating room. It enhances patient safety by ensuring critical steps, identifying risks, and facilitating information exchange. Despite challenges, healthcare organizations should prioritize its implementation to promote a strong safety culture and improve patient outcomes [2].

Perfusionists play a crucial role in cardiothoracic surgeries, operating the heart-lung machine and ensuring vital functions during the procedure. Despite their invaluable contributions, some perfusionists globally may face challenges in voicing concerns during critical moments. Past incidents with insufficient safety devices and monitoring tools led to accidents, but modern cardiopulmonary bypass (CPB) pumps have advanced safety features. Yet, perfusionists may still encounter unwarranted blame for unfavorable outcomes.

The hesitancy to speak up remains a prevalent concern in certain perfusion practices, possibly stemming from the hierarchical structure within the surgical team, fear of being perceived as insubordinate, or the apprehension of facing negative repercussions from fellow team members. However, fostering a robust speak-up culture is essential, empowering all members of the surgical team, including perfusionists, to voice their concerns and observations whenever they notice any issues or potential risks during the procedure [3].

To analyze adverse outcomes and complications thoroughly, conducting debriefs and root cause analyses is crucial. Perfusionists should actively participate, sharing their perspectives to assess issues and contribute valuable suggestions. Implementing inline data management systems is indispensable, protecting perfusionists from unwarranted blame and enabling continuous improvement through retrospective analysis. Promoting a speak-up culture is vital for preventing unsafe practices and fostering an environment of constant improvement and enhanced patient care. Open communication and active participation from all team members can elevate the standards of perfusion care, leading to better outcomes for patients undergoing cardiothoracic surgeries.

Effective communication among perfusionists within the same group or department is crucial for maintaining a cohesive working environment. Monthly team meetings provide an opportunity for collaboration and sharing insights on advancements in the field. Debriefs after difficult cases enable the team to learn from experiences, refine strategies, and improve overall performance. Utilizing modern communication tools further facilitates real-time information exchange. These practices foster a supportive culture, enhancing individual skills and ensuring high-quality patient care, ultimately benefiting the entire department or group.

## Conclusion

Open communication is indispensable in cardiothoracic surgery to ensure patient safety and optimal outcomes. All team members, including perfusionists, should be empowered to voice their concerns and observations. Implementation of the WHO surgical safety checklist and fostering a robust speak-up culture are vital steps towards improving patient care and preventing unsafe practices. By actively engaging in debriefs and utilizing data management systems, continuous improvement can be achieved, leading to better standards of perfusion care and enhanced results for patients undergoing cardiothoracic surgeries.
